# Soybean isoflavones improve the health benefits, flavour quality indicators and physical properties of grass carp (*Ctenopharygodon idella*)

**DOI:** 10.1371/journal.pone.0209570

**Published:** 2019-01-30

**Authors:** Bo Yang, Wei-Dan Jiang, Pei Wu, Yang Liu, Yun-Yun Zeng, Jun Jiang, Sheng-Yao Kuang, Ling Tang, Wu-Neng Tang, Shang-Wen Wang, Xiao-Qiu Zhou, Lin Feng

**Affiliations:** 1 Animal Nutrition Institute, Sichuan Agricultural University, Chengdu, Sichuan, China; 2 Fish Nutrition and Safety Production University Key Laboratory of Sichuan Province, Sichuan Agricultural University, Chengdu, Sichuan, China; 3 Key Laboratory for Animal Disease-Resistance Nutrition of China Ministry of Education, Sichuan Agricultural University, Chengdu, China; 4 Animal Nutrition Institute, Sichuan Academy of Animal Science, Chengdu, Sichuan, China; 5 Tongwei Research Institute, Chengdu, Sichuan, China; University of Illinois, UNITED STATES

## Abstract

Health benefits, flavour quality indicators and physical properties were analysed after feeding grass carp graded concentrations of soybean isoflavones (SIF) (0, 25, 50, 75, 100 and 125 mg/kg) for 60 days. The results demonstrated that optimal dietary SIF supplementation improved the protein and total PUFA content, especially healthcare n-3 PUFA (C18: 3n-3, EPA and DHA), and increased the flavour-related free amino acid [especially umami amino acid] and 5'-inosine monophosphate content, improving the health benefits and flavour quality indicators in the muscle of grass carp. In addition, optimal dietary SIF supplementation (25 or 50 mg SIF/kg diet) enhanced some physical properties [water-holding capacity and tenderness] and increased the collagen content; however, it reduced cathepsin activity and apoptosis. SIF supplementation enhanced the glutathione content and the activity of antioxidant enzymes (except CuZnSOD) by regulating their gene expression. The gene expression could be regulated by NF-E2-related factor 2 (Nrf2) signalling in the muscle of grass carp. We demonstrated that optimal dietary SIF supplementation elevated the health benefits, flavour quality indicators and physical properties of fish muscle.

## Introduction

Fish meat quality has garnered the attention of consumers and the aquaculture industry, since it is directly associated with human health and nutrition [1]. Fish meat quality is composed of a complex set of characteristics, including texture and colour [2], and is heavily influenced by extrinsic factors [3]. Feeding strategy, an important extrinsic factor, is widely used to improve meat quality [4]. Recently, the use of phytogenic feed additives has gained considerable interest to improve meat quality [5–6]. Soybean isoflavones (SIF) are phytogenic additives [7], which are abundant in soybeans [8]. It has been reported that SIF have various biological properties in animals, including anti-estrogenic [9], cardioprotective [10], antifungal [11], antioxidative and anti-inflammatory [12] properties. However, there are only a few reports about the effect of SIF on meat quality. Limited studies have observed that dietary SIF supplementation increased the water-holding capacity (WHC) and improved the colour of male broiler [13]. However, there is a lack of in-depth research on the impact of SIF on meat quality, and whether SIF can influence fish meat quality has not yet been studied.

Meat quality can be evaluated by the fatty acid (FA) profile, which reflects the health benefits of fish [14]. For example, eicosapentaenoic acid (EPA) and docosahexaenoic acid (DHA) are beneficial to humans because they possess the ability to counteract coronary heart disease [15]. However, there is no information about the effect of SIF on the FA profile of fish. Researchers have reported that SIF upregulated Δ6-desaturase (Δ6-D) and stearoyl-CoA desaturase 1 gene expression in the liver of mice [16]. In fish, Δ6-D is the rate-limiting enzyme involved in the biosynthesis of highly unsaturated fatty acids, including EPA and DHA [17]. In addition, Jiang et al [18] showed that stearoyl-CoA desaturase 1 is the rate-limiting enzyme that catalyses the conversion of saturated long-chain FA to monounsaturated fatty acids (MUFA) in mice. These data suggest that SIF might affect the health benefits of animal meat, which awaits investigation.

Apart from FA, free amino acids (FAA) are important indices of meat quality [19]. Because they directly affect taste and participate indirectly in the flavour development of animal meat [20]. For example, glutamic acid (Glu) is not only crucial to the umami taste of animal meat, but also an important aroma precursor in blue mussels [21]. However, there is no available information about the effect of SIF on the FAA profile of animals. In pigs, SIF increased the serum insulin-like growth factor-I (IGF-I) concentration [22], and IGF-I stimulated glutamine absorption in the small intestine [23]. It was reported that glutamine increased the plasma Glu, threonine (Thr), serine (Ser) and glycine (Gly) concentrations in rats [24]. In addition, SIF increased the serum insulin content in rats [25]. Jackim et al. [26] showed that an injection of bovine insulin into *Fundulus heteroclitus* promoted the incorporation of ^14^C-leucine into the muscle. These observations indicate the possibility that SIF might affect the flavour of animal meat, which warrants further investigation.

In addition to FA and FAA, physical properties, such as the water-holding capacity (WHC) and tenderness, are also important quality indicators of fish muscle [27]. To date, evidence that SIF affect the physical properties of fish is lacking. An *in vitro* study showed that SIF could inhibit apoptosis in rat osteoblastic cells [28]. It has been reported that WHC was closely associated with apoptosis in ducks [29]. Moreover, oxidative damage can decrease the tenderness of beef, leading to a decline in meat quality [30]. Generally, oxidative damage is inhibited by NF-E2-related factor 2 (Nrf2)-regulated antioxidative defences in fish [14]. It has been demonstrated that SIF can regulate the Nrf2 signalling pathway in the cerebrovascular tissue of rats [31]. These dada indicated that SIF might influence WHC involved in apoptosis and influence tenderness involved in Nrf2 signaling to change the flesh quality of animals, which could change meat quality, making this topic worthy of investigation.

We investigated the effects of dietary SIF supplementation on the health benefits, flavour quality indicators and physical properties of fish muscle for the first time. This study could reveal evidence of the potential regulatory effect of SIF on the quality of fish. Grass carp, which has a high economic value, is one of the most important freshwater aquaculture species in China [32]. Thus, this study also estimated the optimal SIF supplementation level for grass carp based on growth performance and meat quality parameters. Thus, the results may become an important reference for formulating feeds of grass cap to produce healthier meat.

## Materials and methods

All experimental procedures were approved by the University of Sichuan Agricultural Animal Care Advisory Committee. Grass carp were provided by a fishery (Sichuan, China).

### Experimental diets and procedures

The nutrient levels in the basic diet are shown in Table 1. Fish meal, casein and gelatine were used as the dietary protein sources. Fish oil and corn oil were used as the dietary lipid sources. Soybean isoflavones (SIF, genistin, genistein, daidzin, daidzein, glycitin and glycitein, purity 98%, Shanxi Sciphar Hi-tech Industry Co., Ltd., China) were added to the basic diet to provide graded levels of SIF at 0 (basal diet), 25, 50, 75, 100 and 125 mg SIF/kg feed. All of the dry ingredients in the experimental diets were finely ground and thoroughly mixed together by a mixer until they were homogenous. The mixtures were then made into pellets and air-dried at room temperature. The prepared diets were stored in a freezer for subsequent use according to Zhou et al. [33].

**Table 1:** Composition and nutrients content of basal diet.

Ingredients	%	Nutrient levels	%
**Fish meal**	5.70	**Crude protein**^**4**^	28.67
**Casein**	22.00	**Crude lipid**^**4**^	5.43
**Gelatin**	7.00	**n-3 [67,68]**^**5**^	1.04
**Ca(H**_**2**_**PO**_**4**_**)**_**2**_	1.50	**n-6 [67,68]**^**5**^	0.96
**α-starch**	24.00	**Available phosphorus [68,69]**^**6**^	0.40
**Corn starch**	25.00		
**Fish oil**	2.85		
**Corn oil**	1.58		
**Cellulose**	5.00		
**Vitamin premix** ^**1**^	1.00		
**Mineral premix** ^**2**^	2.00		
**Soybean isoflavones premix**^**3**^	1.00		
**Choline chloride (50%)**	1.00		
**DL-Met (99%)**	0.25		
**L-Trp (99%)**	0.07		
**Ethoxyquin (30%)**	0.05		

^1^ Per kilogram of vitamin premix (g/kg): retinyl acetate (500,000 IU/g), 0.39; cholecalciferol (500,000 IU/g), 0.40; D, L-α-tocopherol acetate (50%), 23.23; menadione (22.9%), 0.83; cyanocobalamin (1%), 0.94; D-biotin (2%), 0.75; folic acid (95%), 0.42; thiamine nitrate (98%), 0.09; ascorhyl acetate (95%), 9.77; niacin (99%), 4.04; meso-inositol (98%), 19.39; calcium-D-pantothenate (98%), 3.85; riboflavin (80%), 0.73; pyridoxine hydrochloride (98%), 0.62. All ingredients were diluted with corn starch to 1 kg.

^2^ Per kilogram of mineral premix (g/kg): MnSO_4_·H_2_O (31.8% Mn), 2.6590; MgSO_4_·H_2_O (15.0% Mg), 200.0000; FeSO_4_·H_2_O (30.0% Fe), 12.2500; ZnSO_4_·H_2_O (34.5% Zn), 8.2460; CuSO_4_·5H_2_O (25.0% Cu), 0.9560; KI (76.9%I), 0.0650; Na_2_SeO_3_ (44.7% Se), 0.0168. All ingredients were diluted with corn starch to 1 kg.

^3^ Soybean isoflavones premix (g/kg): premix was added to obtain graded levels of soybean isoflavones, and the amount of corn starch was reduced to compensate.

^4^ Crude protein and crude lipid contents were measured values.

^5^n-3 and n-6 contents were calculated according to Zeng et al. and calculated according to NRC (2011).

^6^ Available phosphorus were calculated according to Wen et al. and calculated according to NRC (2011).

### Feeding trial

The conditions of the feeding experiment were carried out with reference to a previous study conducted by our laboratory [34]. Grass carp were adapted to the experimental system before initiating the experiment and fed with the basal experimental diet without SIF supplementation for 4 weeks. Next, 540 fish with a mean body weight of 213.78 ± 0.51 g were randomly selected and reared in 18 cages (1.4 L × 1.4 W × 1.4 H m); each cage was stocked with 30 fish according to Wu et al. [35]. Each diet was assigned to three cages. Grass carp were fed with a daily ration of 3%~5% (apparent satiation) of body weight divided into four meals per day for 60 days as described Pan et al. [34]. Uneaten feed was collected using a round plate (diameter 100.0 cm) at the bottom of each cage. The uneaten feed was then dried and weighed to calculate the feed intake (FI) thirty minutes after feeding according to Wu et al. [35]. During the 60-day feeding trial, cages were not cleaned, water temperature and pH were kept constant at 28.5 ± 2.0°C and 7.5 ± 0.3, respectively, and dissolved oxygen was above 6.0 mg/L, nitrite concentration and ammonia concentration were determined to be 0.005–0.010 mg/L and 0.2–0.4 mg/L, respectively. The experimental groups were under a natural light and dark cycle according to Pan et al. [34].

### Sample collection for analysis

Before and after this feeding trial, all grass carp in each cage were harvested and weighed for the calculation of growth-performance-related parameters. Twelve grass carp from each group were quickly caught and anaesthetized with benzocaine before being sacrificed. Then, the muscle slice on the left side above the lateral line and behind the head were immediately removed, quickly frozen in liquid nitrogen and stored at -80°C until analysis according to Salmerón et al. [36]. Meanwhile, the muscle on the other side above the lateral line and behind the head was removed for analysis of physical properties (shear force and cooking loss) as described by Wu et al. [35]. Briefly, the muscle samples were heat-sealed in duplicates in PE-bags and heated at 70°C for 20 min. After cooling, the cooking loss was calculated. Then, the samples were placed in a shear box and the relative shear force of the cooked flesh was measured. The moisture, crude protein and lipid content were measured following the methods of Jiang et al. [14]. Briefly, samples of diet and fish muscle were dried to a constant weight at 105°C to measure moisture; protein of fish muscle was determined by measuring nitrogen (N×6.25) using the Kjeldahl method; lipid of fillet was obtained by ether extraction using Soxhlet. The analysis of crude protein and crude lipid content in feed was similar to the fish muscle. The hydroxyproline content and the cathepsin B and L activities were measured according to the procedures described in our previous study [14]. The FAA composition and the 5'-inosine monophosphate (5'-IMP) content were measured in accordance with a previous study [37]. The FA composition of the fish muscle was measured using gas chromatography according to our previous study Jiang et al. [14].

The fish samples were weighed (0.4 g) and homogenized in ice-cold physiological saline at the proportion of 1:9 (w/v). Then, the samples were centrifuged (6000 ***g***, 20 min, 4°C), and the supernatants were used to quantify the antioxidant capacity. The reactive oxygen species (ROS), malondialdehyde (MDA), protein carbonyl (PC) and glutathione (GSH) content, and the zinc superoxide dismutase (CuZnSOD), manganese superoxide dismutase (MnSOD), glutathione peroxidase (GPx), catalase (CAT), glutathione-transferase (GST) and glutathione reductase (GR) activities were assayed according to the instruction in the kits purchased from Nanjing Jiancheng Bioengineering Institute China.

### Analysis of DNA fragmentation

The assay for apoptotic cell fragmentation was conducted following our previous study [38]. Agarose gel electrophoresis was used to analyse DNA fragmentation. The DNA was loaded into a 2.0% agarose gel, and then electrophoresis was conducted at 40 V for 1.5 h. A Gene Genius Bio-Imaging system (Syngene, Frederick, MD, USA) was used to examine and photograph the gel.

### Real-time PCR analysis

Real-time PCR was used to determine the relative quantity of antioxidant and apoptosis-related target genes. Briefly, total RNA of the muscle was extracted using an RNAiso Plus Kit (Takara, Dalian, China) following the manufacturer’s instructions. Then, agarose gel (1%) electrophoresis and spectrophotometry (A260: 280 nm ratio) were used to measure RNA quantity and purity, respectively. Subsequently, RNA was reverse transcribed into cDNA using a PrimeScript RT Reagent Kit (TaKaRa). For quantitative real-time PCR, specific primers were designed according to the sequences cloned in our lab and the published sequences of grass carp in NCBI (Table 2). According to our first experiment concerning the evaluation of internal control genes, *β-actin* was used as a reference gene to normalize cDNA loading (data did not show here). The target and housekeeping gene amplification efficiencies were calculated following specific gene standard curves generated from 10-fold serial dilutions. The expression results were calculated using the 2^-ΔΔCT^ method as described by Livak et al. [39].

**Table 2:** Real-time PCR primer sequences ^1^.

Target gene	Primer sequence Forward (5’→3’)	Primer sequence Reverse (5’→3’)	Temperature(°C)	Accession number
***Δ6-D***	ATGCTCAATGCGTTTGTAGT	AGGAGGTCCAATGAAGAAGA	63.0	AY445923
***caspase-2***	CGCTGTTGTGTGTTTACTGTCTCA	ACGCCATTATCCATCTCCTCTC	60.3	KT757313
***caspase-3***	GCTGTGCTTCATTTGTTTG	TCTGAGATGTTATGGCTGTC	55.9	JQ793789
***caspase-7***	GCCATTACAGGATTGTTTCACC	CCTTATCTGTGCCATTGCGT	57.1	KT625601
***caspase-8***	ATCTGGTTGAAATCCGTGAA	TCCATCTGATGCCCATACAC	59.0	KM016991
***caspase-9***	CTGTGGCGGAGGTGAGAA	GTGCTGGAGGACATGGGAAT	59.0	JQ793787
***CuZnSOD***	CGCACTTCAACCCTTACA	ACTTTCCTCATTGCCTCC	61.5	GU901214
***MnSOD***	ACGACCCAAGTCTCCCTA	ACCCTGTGGTTCTCCTCC	60.4	GU218534
***CAT***	GAAGTTCTACACCGATGAGG	CCAGAAATCCCAAACCAT	58.7	FJ560431
***GPx1a***	GGGCTGGTTATTCTGGGC	AGGCGATGTCATTCCTGTTC	61.5	EU828796
***GPx1b***	TTTTGTCCTTGAAGTATGTCCGTC	GGGTCGTTCATAAAGGGCATT	60.3	KT757315
***GPx4a***	TACGCTGAGAGAGGTTTACACAT	CTTTTCCATTGGGTTGTTCC	60.4	KU255598
***GPx4b***	CTGGAGAAATACAGGGGTTACG	CTCCTGCTTTCCGAACTGGT	60.3	KU255599
***GSTR***	TCTCAAGGAACCCGTCTG	CCAAGTATCCGTCCCACA	58.4	EU107283
***GSTP1***	ACAGTTGCCCAAGTTCCAG	CCTCACAGTCGTTTTTTCCA	59.3	KM112099
***GSTP2***	TGCCTTGAAGATTATGCTGG	GCTGGCTTTTATTTCACCCT	59.3	KP125490
***GSTO1***	GGTGCTCAATGCCAAGGGAA	CTCAAACGGGTCGGATGGAA	58.4	KT757314
***GSTO2***	CTGCTCCCATCAGACCCATTT	TCTCCCCTTTTCTTGCCCATA	61.4	KU245630
***GR***	GTGTCCAACTTCTCCTGTG	ACTCTGGGGTCCAAAACG	59.4	JX854448
***Nrf2***	CTGGACGAGGAGACTGGA	ATCTGTGGTAGGTGGAAC	62.5	KF733814
***Keap1a***	TTCCACGCCCTCCTCAA	TGTACCCTCCCGCTATG	63.0	KF811013
***Keap1b***	TCTGCTGTATGCGGTGGGC	CTCCTCCATTCATCTTTCTCG	57.9	KJ729125
***TOR***	TCCCACTTTCCACCAACT	ACACCTCCACCTTCTCCA	61.4	JX854449
***S6K1***	GCAATCTGCTGAGGATGTGA	AACCGACGAGGTGAACGA	56.4	KY939577
***4E-BP1***	GCTGGCTGAGTTTGTGGTTG	CGAGTCGTGCTAAAAAGGGTC	60.3	KT757305
***4E-BP2***	CACTTTATTCTCCACCACCCC	TTCATTGAGGATGTTCTTGCC	60.3	KT757306
***β-actin***	GGCTGTGCTGTCCCTGTA	GGGCATAACCCTCGTAGAT	61.4	M25013

^1^*Δ6-D*: *Δ*6-desaturase; *caspase*, cysteinyl aspartic acid-protease; *CuZnSOD*, copper/zinc superoxide dismutase; *MnSOD*, manganese superoxide dismutase; *CAT*, catalase; *GPx*, glutathione peroxidase; *GST*, glutathione-*S*-transferase; *GR*, glutathione reductase; N*rf2*, NF-E2-related factor 2; *Keap1*, Kelch-like-ECH-associated protein 1; *TOR*, target of rapamycin; *S6K1*, ribosomal protein S6 kinase 1; *4E-BP*, eIF4E-binding protein

### Western blotting

The processes for muscle protein extract preparation, antibodies and western blotting were conducted according to our previous research [40]. In brief, we determined the protein concentrations using a BCA assay kit purchased from the Beyotime Institute of Biotechnology (Shanghai, China). An amount of protein was subjected to 10% SDS-PAGE and transferred onto a PVDF membrane (purchased Millipore, Cheng Du, China), blocked for 1 h at room temperature (25–28°C) and then incubated overnight with the primary antibody at 4°C. We used the same anti-total TOR, p-TOR Ser 2448, Nrf2, β-actin and lamin B1 antibodies as described in our previous research [40]. β-actin and lamin B1 were used as control proteins for total and nuclear protein, respectively. After washing three times, the PVDF membranes were incubated with the HRP-conjugated secondary antibody in TBST for 2 h at room temperature. Immune complexes were visualized using an ECL kit (Millipore). NIH Image 1.63 software was used to quantify the density of the western bands. The results for all protein levels of the densitometric analyses were expressed as the fold of SIF treatment groups relative to the non-supplemented group. The western blot results from each group were measured three times independently.

### Calculations and statistical analysis

The formulas for growth performance parameters, percentage weight gain (PWG), specific growth rate (SGR) and feed efficiency (FE), of grass carp were calculated using initial body weight (IBW), final body weight (FBW) and feed intake (FI).

PWG=100×[FBW(g/fish)−IBW(g/fish)]/IBW(g/fish)

FE=100×[FBW(g/fish)−IBW(g/fish)]/FI(g/fish)

SGR=100×[In(finalbodyweight)−In(initialbodyweight)]/days.

The results were expressed as the mean values ± SD. All data were analysed by a one-way analysis of variance followed by Duncan’s multiple range tests to determine significant differences among the 6 treatment groups using SPSS 18.0 (SPSS Inc., Chicago, IL, USA). Significance was declared at *P* < 0.05.

## Results

### Effects of SIF on the fish health, growth performance and muscle composition of grass carp

No diet related mortalities or external macroscopic alterations attributable to any of the dietary treatments were found. As shown in Table 3, FBW, PWG, SGR, FI and FE of the grass carp fed the 25 mg/kg SIF diet were significantly higher than those of the non-supplemented group (*P* < 0.05). The protein, lipid and calcium content of the fish muscle reached a maximum when grass carp were supplemented with SIF at 50 mg/kg, and they gradually decreased as the SIF level further increased. The moisture content reached a minimum at 50 mg SIF/kg feed, and it slowly increased thereafter. However, supplementation of SIF did not have a significant effect on the phosphorus and ash content (*P* > 0.05).

**Table 3:** Growth performance and muscle composition of grass carp fed the diets with graded level of SIF for 60 days^1^.

	Dietary SIF levels(mg/kg diet)						
	0		25	50	75	100	125
**IBW**^**1**^	213.78 ± 0.77^a^	213.56 ± 0.38^a^		214.22 ± 0.38^a^	213.56 ± 0.38^a^	213.78 ± 0.77^a^	213.78 ± 0.38^a^
**FBW**^**1**^	755.52 ± 53.88^b^		911.36 ±127.7^d^	849.48 ± 37.66^c^	817.3 ± 68.65^c^	729.05 ± 32.07^b^	645.98 ± 47.82^a^
**PWG**^**1**^	253.43 ± 8.44^b^		323.10 ± 11.56^d^	294.54 ± 9.48^c^	283.09 ± 43.70^c^	243.50 ± 14.09^b^	202.70 ± 7.96^a^
**SGR**^**1**^	2.1 ± 0.02^b^		2.4 ± 0.02^d^	2.29 ± 0.02^c^	2.24 ± 0.09^c^	2.06 ± 0.04^b^	1.85 ± 0.02^a^
**FI**^**1**^	848.66 ± 0.69^c^		957.39 ± 1.68^f^	915.21 ± 2.06^e^	854.51 ± 1.18^d^	781.69 ± 3.31^b^	711.52 ± 2.13^a^
**FE**^**1**^	63.84 ± 1.04^ab^		72.07 ± 1.25^d^	68.94 ± 1.09^cd^	70.75 ± 5.16^cd^	66.59 ± 1.54^bc^	60.90 ± 0.94^a^
**Moisture (%)**^**2**^	79.21 ± 0.37^c^		78.02 ± 0.89^ab^	77.14 ± 0.33^a^	77.84 ± 0.43^a^	78.85 ± 0.77^bc^	79.44 ± 1.43^c^
**Protein (%)**^**2**^	16.00 ± 0.59^a^		17.44 ± 0.49^cd^	17.91 ± 0.34^d^	17.36 ± 0.66^cd^	16.81 ± 0.69^bc^	16.50 ± 0.93^ab^
**Lipid (%)**^**2**^	3.05 ± 0.22^b^		3.34 ± 0.31^bc^	3.58 ± 0.13^c^	3.43 ± 0.29^c^	3.10 ± 0.25^b^	2.67 ± 0.27^a^
**Ash (%)**^**2**^	1.07 ± 0.11^a^		0.97 ± 0.10^a^	0.98 ± 0.09^a^	0.99 ± 0.08^a^	0.97 ± 0.07^a^	0.98 ± 0.09^a^
**Calcium**	0.75 ± 0.06^a^		1.00 ± 0.03^bc^	1.06 ± 0.09^c^	0.97 ± 0.06^b^	0.80 ± 0.04^a^	0.81 ± 0.08^a^
**Phosphorus**	0.29 ± 0.01^a^		0.31 ± 0.01^a^	0.31 ± 0.01^a^	0.30 ± 0.02^a^	0.30 ± 0.02^a^	0.31 ± 0.01^a^

^1^ IBW: Initial body weight (g/fish); FBW: final body weight (g/fish); PWG: percent weight gain (%); SGR: specific growth rate (%/day); FI: feed intake (g/fish); FE: feed efficiency (%);Values are means ± SD for three replicate groups, with 30 fish in each group, and different superscripts in the same row are significantly different (*P* < 0.05).

^2^ Values are means ± SD (n = 6), and different superscripts in the same row are significantly different (*P* < 0.05).

### Effects of SIF on the meat quality parameters of grass carp

The changes in the FA profile and in *Δ6-D* gene expression caused by dietary SIF supplementation are summarized in Table 4. Compared with the non-supplemented group, the group fed the 50 mg/kg SIF diet had a lower C16:0 and C20:1n9 fatty acid content. The C22:2 content peaked at 25 mg SIF/kg feed (*P* < 0.05) and then slowly decreased. The C18:3n-3(ALA**)**, EPA and DHA content reached a maximum at 50 mg SIF/kg feed and slowly declined thereafter. A lower content of total SFA was obtained when the fish were supplemented with 50 mg SIF/kg feed than with the other supplementation levels. Conversely, the highest content of total unsaturated fatty acid and total PUFA were obtained with the 50 mg/kg SIF treatment. Nevertheless, the content of other FA were not affected by SIF supplementation (*P* > 0.05). ∑n3/∑n6 reached a maximum when grass carp were supplemented with SIF at 50 mg/kg, and then gradually decreased as the SIF level further increased. The gene expression of *Δ6-D* was elevated with dietary SIF supplementation; it increased to the 50 mg/kg SIF level and decreased thereafter.

**Table 4:** Effects of dietary SIF supplementation (mg/kg) on muscle fillet fatty acid composition (% total fatty acids) and *Δ*6-desaturase gene expression of grass carp^1^.

	Dietary SIF levels(mg/kg diet)					
	0	25	50	75	100	125
**C14: 0**	2.40 ± 0.15^a^	2.32 ± 0.21^a^	2.26 ± 0.20^a^	2.34 ± 0.22^a^	2.32 ± 0.22^a^	2.50 ± 0.17^a^
**C15: 0**	0.25 ± 0.03^a^	0.24 ± 0.02^a^	0.23 ± 0.02^a^	0.23 ± 0.04^a^	0.22 ± 0.01^a^	0.25 ± 0.03^a^
**C16: 0**	25.23 ± 0.54^b^	23.60 ± 1.21^ab^	21.45 ± 1.87^a^	23.78 ± 1.10^ab^	24.48 ± 1.45^b^	24.46 ± 1.56^b^
**C17: 0**	0.16 ± 0.01^a^	0.15 ± 0.02^a^	0.14 ± 0.02^a^	0.14 ± 0.01^a^	0.15 ± 0.02^a^	0.16 ± 0.01^a^
**C18: 0**	3.76 ± 0.27^a^	3.62 ± 0.40^a^	3.74 ± 0.21^a^	3.71 ± 0.46^a^	3.64 ± 0.44^a^	3.74 ± 0.43^a^
**C20: 0**	0.19 ± 0.02^a^	0.19 ± 0.01^a^	0.17 ± 0.03^a^	0.19 ± 0.02^a^	0.18 ± 0.01^a^	0.21 ± 0.03^a^
**C21: 0**	0.08 ± 0.00^a^	0.07 ± 0.01^a^	0.07 ± 0.01^a^	0.07 ± 0.01^a^	0.08 ± 0.01^a^	0.08 ± 0.01^a^
**C22: 0**	0.58 ± 0.07^a^	0.58 ± 0.06^a^	0.56 ± 0.06^a^	0.58 ± 0.07^a^	0.59 ± 0.09^a^	0.63 ± 0.01^a^
**C23: 0**	0.79 ± 0.03^a^	0.75 ± 0.06^a^	0.76 ± 0.08^a^	0.76 ± 0.03^a^	0.77 ± 0.08^a^	0.81 ± 0.12^a^
**C14: 1**	0.15 ± 0.00^a^	0.15 ± 0.01^a^	0.15 ± 0.01^a^	0.15 ± 0.01^a^	0.15 ± 0.01^a^	0.16 ± 0.01^a^
**C16: 1**	11.13 ± 0.98^a^	10.31 ± 0.83^a^	10.21 ± 1.35^a^	10.54 ± 1.25^a^	10.71 ± 1.14^a^	11.63 ± 1.66^a^
**C17: 1**	0.27 ± 0.04^a^	0.26 ± 0.04^a^	0.25 ± 0.04^a^	0.25 ± 0.04^a^	0.27 ± 0.03^a^	0.30 ± 0.04^a^
**C18:1n9t**	0.26 ± 0.03^a^	0.25 ± 0.02^a^	0.25 ± 0.03^a^	0.26 ± 0.02^a^	0.24 ± 0.03^a^	0.26 ± 0.03^a^
**C18:1n9c**	32.85 ± 1.02^a^	34.35 ± 1.16^a^	36.11 ± 2.36^a^	35.32 ± 1.04^a^	34.87 ± 0.70^a^	33.50 ± 2.94^a^
**C20:1n9**	1.94 ± 0.14^b^	1.69 ± 0.12^ab^	1.52 ± 0.10^a^	1.53 ± 0.20^a^	1.79 ± 0.17^ab^	1.92 ± 0.31^b^
**C22: 1n-9**	0.05 ± 0.01^a^	0.05 ± 0.01^a^	0.05 ± 0.01^a^	0.05 ± 0.00^a^	0.05 ± 0.00^a^	0.05 ± 0.00^a^
**C24: 1n-9**	0.04 ± 0.00^a^	0.04 ± 0.00^a^	0.04 ± 0.01^a^	0.04 ± 0.00^a^	0.04 ± 0.00^a^	0.04 ± 0.01^a^
**C18:2n6t**	0.03 ± 0.00^a^	0.03 ± 0.00^a^	0.03 ± 0.00^a^	0.03 ± 0.00^a^	0.03 ± 0.01^a^	0.04 ± 0.00^a^
**C18:2n6c**	10.33 ± 1.20^a^	10.08 ± 1.47^a^	10.07 ± 0.77^a^	9.25 ± 0.99^a^	9.62 ± 0.26^a^	10.58 ± 0.80^a^
**C20: 2**	0.07 ± 0.01^a^	0.07 ± 0.01^a^	0.07 ± 0.00^a^	0.07 ± 0.00^a^	0.07 ± 0.01^a^	0.07 ± 0.01^a^
**C22: 2**	0.12 ± 0.01^ab^	0.14 ± 0.02^c^	0.13 ± 0.01^ab^	0.11 ± 0.01^a^	0.11 ± 0.01^a^	0.12 ± 0.00^ab^
**C18: 3n-6**	0.10 ± 0.01^a^	0.10 ± 0.01^a^	0.10 ± 0.01^a^	0.10 ± 0.01^a^	0.10 ± 0.01^a^	0.11 ± 0.02^a^
**C18: 3n-3(ALA)**	0.58 ± 0.01^a^	0.65 ± 0.07^a^	1.00 ± 0.09^b^	0.89 ± 0.10^b^	0.57 ± 0.09^a^	0.61 ± 0.05^a^
**C20: 3n-6**	0.37 ± 0.01^a^	0.37 ± 0.03^a^	0.39 ± 0.03^a^	0.37 ± 0.03^a^	0.36 ± 0.02^a^	0.38 ± 0.01^a^
**C20: 3n-3**	0.06 ± 0.00^a^	0.06 ± 0.00^a^	0.06 ± 0.00^a^	0.06 ± 0.01^a^	0.06 ± 0.01^a^	0.07 ± 0.00^a^
**C20: 5n-3(EPA)**	1.40 ± 0.07^a^	1.64 ± 0.08^bc^	1.72 ± 0.10^c^	1.53 ± 0.07^b^	1.38 ± 0.04^a^	1.35 ± 0.04^a^
**C22: 6n-3(DHA)**	6.80 ± 0.24^ab^	8.23 ± 0.78^b^	8.46 ± 1.16^b^	7.64 ± 1.00^b^	7.15 ± 0.97^ab^	5.96 ± 0.66^a^
**SFA**	33.43 ± 0.39^b^	31.53 ± 1.03^ab^	29.38 ± 1.55^a^	31.79 ± 0.36^b^	32.42 ± 0.78^b^	32.85 ± 2.32^b^
**UFA**	66.57 ± 0.39^a^	68.47 ± 1.03^ab^	70.62 ± 1.55^b^	68.21 ± 0.36^a^	67.58 ± 0.78^a^	67.15 ± 2.32^a^
**MUFA**	46.70 ± 1.29^a^	47.09 ± 1.64^a^	48.59 ± 3.15^a^	48.14 ± 1.55^a^	48.12 ± 1.67^a^	47.85 ± 0.95^a^
**PUFA**	19.88 ± 0.91^ab^	21.37 ± 0.67^ab^	22.03 ± 1.81^b^	20.07 ± 1.29^ab^	19.46 ± 1.14^a^	19.30 ± 1.39^a^
∑n3/∑n6	0.81±0.13^ab^	0.99±0.23^b^	1.06±0.09^b^	1.03±0.14^b^	0.90±0.07^ab^	0.72±0.01^a^
*Δ****6-D***	1.03 ± 0.25^a^	1.30 ± 0.32^ab^	1.62 ± 0.26^b^	1.32 ± 0.36^ab^	1.07 ± 0.18^ab^	1.05 ± 0.18^a^

^1^All data were expressed as means ± SD (n = 6). Mean values within the same row with different superscripts are significantly different (*P* < 0.05); SFA, saturated fatty acid; MUFA, monounsaturated fatty acid; PUFA, polyunsaturated fatty acid; *Δ6-D*, *Δ*6-desaturase gene.

**Fig 1:**
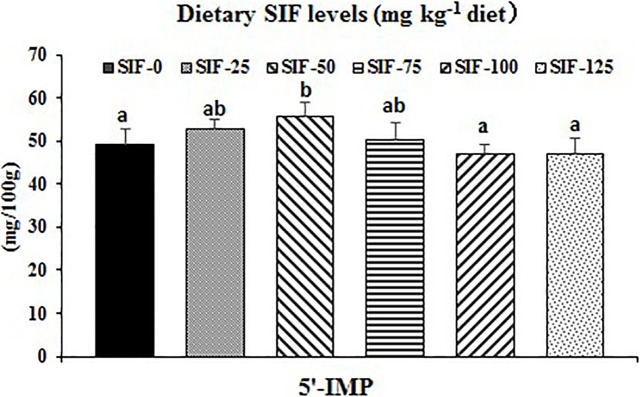
Effects of SIF on the 5'-IMP in the muscle of grass carp. All data were expressed as means ± SD (n = 6). Mean values within the same row with different superscripts are significantly different (*P* < 0.05). 5'-IMP, 5'-inosine monophosphate.

Effects of dietary SIF supplementation on FAA are presented in Table 5. The Glu, Asp, Ser, Gly, alanine (Ala), lysine (Lys), leucine (Leu), isoleucine (IIe), arginine (Arg) and total FAA content all reached a maximum value with the 50 mg /kg SIF treatment. The phenylalanine (Phe) and cysteine (Cys) content increased with dietary SIF supplementation, increasing to 25 mg SIF/kg feed (*P* < 0.05) and decreasing thereafter. The tyrosine (Tyr) content decreased, reached a minimum at the 50 mg/kg SIF supplementation level, and slowly increased thereafter. However, dietary SIF supplementation did not significantly affect the methionine (Met), Thr, valine (Val) and histidine (His) content (*P* > 0.05). As presented in Fig 1, compared with non-supplemented group, the5'-IMP content was up-regulated with dietary SIF supplementation levels increased to 50 mg/kg diet, and then and gradually down-regulated.

**Table 5:** Effects of dietary SIF supplementation (mg/kg) on muscle amino acid composition (mg/100 g tissue) of grass carp^1^.

	Dietary SIF levels(mg/kg diet)					
	0	25	50	75	100	125
**Glu**	9.22 ± 0.62^ab^	11.16 ± 0.82^c^	11.89 ± 0.88^c^	11.28 ± 1.32^c^	10.52 ± 0.79^bc^	8.59 ± 0.86^a^
**Asp**	1.54 ± 0.05^ab^	1.67 ± 0.13^b^	1.69 ± 0.05^b^	1.65 ± 0.08^ab^	1.61 ± 0.06^ab^	1.51 ± 0.08^a^
**Ser**	8.98 ± 0.74^a^	9.11 ± 0.52^ab^	10.38 ± 1.17^b^	10.10 ± 0.48^ab^	9.18 ± 0.33^ab^	8.97 ± 0.71^a^
**Gly**	85.14 ± 1.28^a^	89.53 ± 1.89^ab^	92.22 ± 3.57^b^	89.45 ± 1.42^ab^	85.50 ± 2.22^a^	85.32 ± 4.43^a^
**Ala**	21.92 ± 0.73^a^	25.59 ± 1.75^b^	26.26 ± 0.47^b^	22.04 ± 0.73^a^	21.71 ± 0.87^a^	21.68 ± 1.38^a^
**Lys**	15.37 ± 0.85^a^	17.26 ± 2.16^ab^	20.53 ± 1.69^c^	19.81 ± 1.93^bc^	18.82 ± 1.42^bc^	18.53 ± 1.61^bc^
**Met**	3.74 ± 0.18^a^	3.50 ± 0.29^a^	3.39 ± 0.06^a^	3.48 ± 0.33^a^	3.40 ± 0.24^a^	3.50 ± 0.35^a^
**Thr**	15.61 ± 0.94^a^	14.99 ± 0.85^a^	15.99 ± 1.24^a^	14.73 ± 0.95^a^	14.54 ± 1.39^a^	14.15 ± 0.45^a^
**Leu**	4.64 ± 0.24^a^	5.02 ± 0.45^ab^	5.49 ± 0.47^b^	5.25 ± 0.28^ab^	4.79 ± 0.27^a^	4.64 ± 0.33^a^
**Ile**	2.95 ± 0.33^a^	3.23 ± 0.30^ab^	3.50 ± 0.22^b^	2.87 ± 0.15^a^	2.83 ± 0.20^a^	3.03 ± 0.23^a^
**Arg**	17.02 ± 1.56^ab^	17.54 ± 0.92^ab^	18.50 ± 1.61^b^	18.27 ± 1.93^b^	15.79 ± 1.05^ab^	14.93 ± 1.40^a^
**Val**	5.18 ± 0.42^a^	5.26 ± 0.28^a^	5.30 ± 0.53^a^	5.07 ± 0.23^a^	5.05 ± 0.35^a^	4.96 ± 0.69^a^
**Phe**	3.72 ± 0.10^a^	5.19 ± 0.37^c^	4.92 ± 0.32^c^	4.36 ± 0.21^b^	4.16 ± 0.45^ab^	3.71 ± 0.29^a^
**His**	175.82 ± 11.29^a^	185.56 ± 8.61^a^	177.72 ± 7.87^a^	171.74 ± 6.35^a^	172.84 ± 6.83^a^	169.18 ± 9.60^a^
**Cys**	0.44 ± 0.02^a^	0.58 ± 0.05^b^	0.56 ± 0.04^b^	0.45 ± 0.02^a^	0.44 ± 0.04^a^	0.41 ± 0.04^a^
**Tyr**	3.72 ± 0.22^b^	3.39 ± 0.22^ab^	3.07 ± 0.37^a^	3.42 ± 0.17^ab^	4.88 ± 0.30^c^	4.69 ± 0.36^c^
**Total**	375.00 ± 12.97^a^	398.58 ± 4.76^b^	401.39 ± 13.53^b^	383.81 ± 5.32^ab^	376.08 ± 8.41^a^	367.78 ± 12.83^a^

^1^All data were expressed as means ± SD (n = 6). Mean values within the same row with different superscripts are significantly different (*P* < 0.05).

As presented in Table 6, relative shear force and cooking loss reached a minimum at the 50 mg/kg SIF level, and gradually increased thereafter. Cathepsin (B and L) activity reached a minimum when grass carp were supplemented with SIF at 50 mg/kg, and they gradually increased as the SIF level increased. The highest hydroxyproline content was obtained with the 50 mg/kg SIF treatment.

**Table 6:** Muscle shear force (N), cooking loss (%), hydroxyproline concentration (mg/g tissue) and cathepsin B and L activities (U/g muscle) of grass carp fed diets with graded levels of SIF (mg/kg)^1^.

	Dietary SIF levels(mg/kg diet)					
	0	25	50	75	100	125
**Shear force**	1.25 ± 0.38^b^	1.17 ± 0.61^ab^	1.16± 0.43^a^	1.18 ± 0.53^ab^	1.20 ± 0.83^ab^	1.22 ± 0.60^ab^
**Cooking loss**	14.38 ± 0.79^b^	13.41 ± 0.86^ab^	13.11 ± 0.80^a^	13.55 ± 1.24^ab^	14.12 ± 1.02^ab^	14.32 ± 0.68^b^
**Hydroxyproline**	0.48 ± 0.04^b^	0.5 7 ± 0.04^d^	0.5 9 ± 0.03^d^	0.53 ± 0.01^c^	0.43 ± 0.03^a^	0.40 ± 0.02^a^
**Cathepsin B**	3.89 ± 0.22^cd^	3.18 ± 0.22^ab^	3.09 ± 0.25^a^	3.42 ± 0.25^b^	3.75 ± 0.25^c^	4.13 ± 0.14^d^
**Cathepsin L**	2.02 ± 0.12^c^	1.67 ± 0.13^a^	1.66 ± 0.04^a^	1.81 ± 0.15^ab^	1.95 ± 0.17^bc^	2.20 ± 0.04^d^

^1^All data were expressed as means ± SD (n = 6). Mean values within the same row with different superscripts are significantly different (*P* < 0.05).

### Effects of SIF on apoptosis in fish muscle

**Fig 2:**
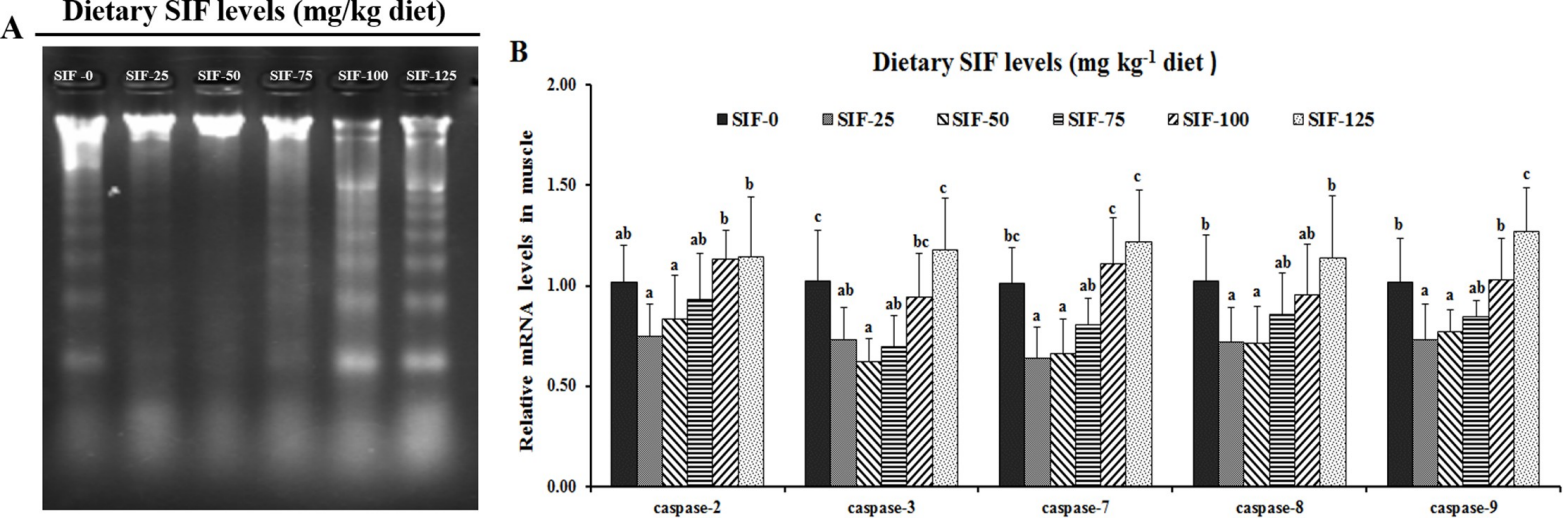
Effects of SIF on the apoptosis in the muscle of grass carp. **(A) DNA fragmentation. (B) *Caspase-2*, *-3*, *-7*, *-8* and *-9* genes expression.** Values are means (n = 6 for gene expression), error bars indicate SD, and different letters above a bar denote the significant difference between treatments (*P* < 0.05). caspase, cysteinyl aspartic acid-protease.

The effect of dietary SIF supplementation on DNA fragmentation is presented in Fig 2A. Compared with the non-supplemented group, DNA fragmentation was decreased with dietary SIF supplementation levels increased to 50 mg/kg diet, and then gradually increased.

The mRNA expression of genes involved in apoptosis was determined. As presented in Fig 2B, the mRNA levels o*f caspase-2*, *caspase-3* and *caspase-8* were affected by supplementation and obtained minimum values at 25, 50 and 50 mg SIF/kg feed, respectively. The mRNA levels of *caspase-7* and *caspase-9* significantly differed among all treatments (*P* < 0.05) and were lowest with the 50 mg/kg SIF treatment.

### Effects of SIFs on antioxidant-related parameters, mRNA expression and related signalling molecules in fish muscle

The effects of dietary SIF supplementation on antioxidant-related parameters are shown in Table 7. The MDA content decreased, reached a minimum at 50 mg SIF/kg feed, and slowly increased thereafter. The PC content decreased, reached a minimum at 50 mg SIF/kg feed, and slowly increased thereafter. The ROS content reached a minimum value when grass carp were supplemented 50 mg SIF/kg feed and gradually increased with higher levels of supplementation. The highest activities of MnSOD, CAT, GPx and GR were obtained with the 50 mg/kg SIF treatment. The activity of GST and the GSH content significantly increased until they reached a maximum value at 50 mg SIF/kg feed (*P* < 0.05), and then they slowly declined thereafter. However, supplementation of SIF had no significant effect on the activity of CuZnSOD (*P* > 0.05).

**Table 7:** Effects of dietary SIF supplementation (mg/kg) on antioxidant parameters in the muscle of grass carp^1^.

	Dietary SIF levels(mg/kg diet)					
	0	25	50	75	100	125
**ROS**	99.91 ± 7.78^d^	79.81 ± 5.07^ab^	77.20 ± 4.23^a^	85.48 ±3.79^bc^	88.14 ± 5.01^c^	97.81 ± 8.65^d^
**MDA**	11.02±0.58^b^	9.86±0.33^a^	9.16±0.88^a^	10.01±0.73^a^	11.57±1.04^b^	13.14±0.23^c^
**PC**	2.49 ± 0.13^c^	1.47 ± 0.13^a^	1.42 ± 0.16^a^	1.53 ± 0.12^a^	1.80 ± 0.15^b^	2.48 ± 0.18^c^
**CuZnSOD**	2.48 ± 0.15^a^	2.4 8± 0.09^a^	2.57 ± 0.26^a^	2.55 ± 0.15^a^	2.52 ± 0.12^a^	2.48 ± 0.14^a^
**MnSOD**	2.99 ± 0.20^ab^	3.39 ± 0.31^bc^	3.79 ± 0.07^c^	3.58 ± 0.29^c^	2.72 ± 0.54^a^	2.71 ± 0.39^a^
**CAT**	2.21 ± 0.09^a^	3.99 ± 0.27^cd^	4.21 ± 0.33^d^	3.91 ± 0.22^bc^	3.64 ± 0.31^b^	2.00 ± 0.14^a^
**GPx**	139.87 ± 7.25^ab^	151.19 ± 14.06^bc^	167.96 ± 11.54^d^	166.94 ± 14.76^d^	156.82 ± 8.09^bc^	133.34 ± 6.85^a^
**GST**	61.06 ± 5.56^a^	76.60 ± 7.05^b^	93.99 ± 7.59^c^	80.42 ± 5.03^b^	62.95 ± 5.37^a^	57.03 ± 4.85^a^
**GR**	23.65 ± 1.70^b^	26.34 ± 2.27^c^	28.21 ± 1.89^c^	26.68 ± 1.80^c^	23.84 ± 1.90^b^	20.96 ± 1.67^a^
**GSH**	3.52 ± 0.30^a^	4.15 ± 0.32^b^	4.51 ± 0.29^c^	4.27 ± 0.26^bc^	4.09 ± 0.27^b^	3.34 ± 0.18^a^

^1^All data were expressed as means ± SD (n = 6). Mean values within the same row with different superscripts are significantly different (*P* < 0.05). ROS, reactive oxygen species (% DCF florescence); MDA, malondialdehyde (nmol/g tissue); PC, protein carbonyl (nmol/mg protein); CuZnSOD, copper/zinc superoxide dismutase (U/mg protein); MnSOD, manganese superoxide dismutase (U/mg protein); CAT, catalase (U/mg protein); GPx, glutathione peroxidase (U/mg protein); GST, glutathione-*S*-transferase (U/mg protein); GR, glutathione reductase (U/g protein); GSH, glutathione (mg/g protein).

**Fig 3:**
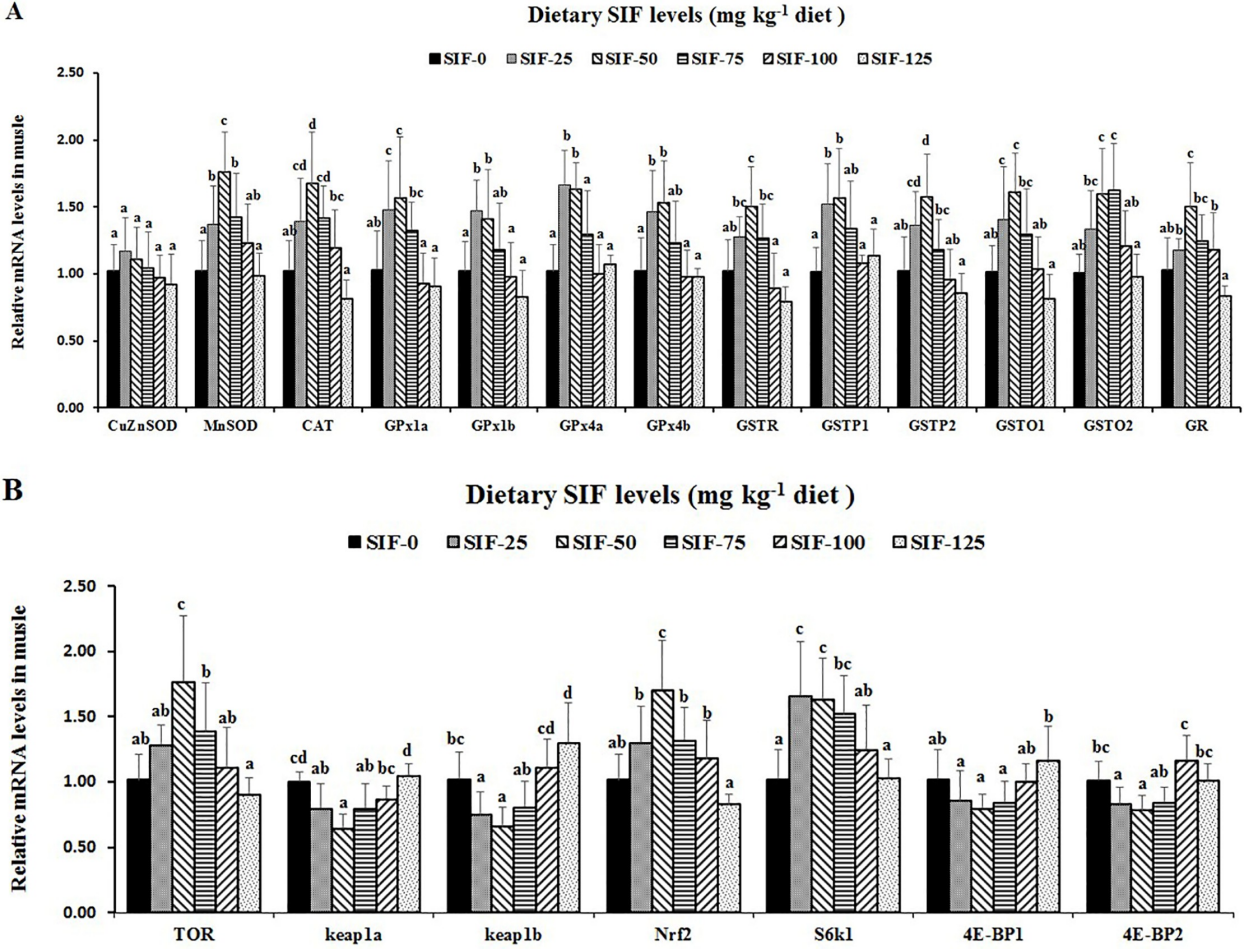
Effects of SIF on the antioxidative enzyme genes expression and signaling molecules in the muscle of grass carp. **(A) *CuZnSOD*, *MnSOD*, *CAT*, *GPx1a*, *GPx1b*, *GPx4a*, *GPx4b*, *GSTR*, *GSTP1*, *GSTP2*, *GSTO1*, *GSTO2* and *GR*. (B) *TOR*, *Keap1a*, *Keap1b*, *Nrf2*, *S6K1*, *4E-BP1* and *4E-BP2*.** Values are means (n = 6 for gene expression), error bars indicate SD, and different letters above a bar denote the significant difference between treatments (*P* < 0.05).

The mRNA levels of antioxidant-related enzymes and signalling molecules were measured. As shown in Fig 3A and 3B, the mRNA level of *MnSOD* significantly increased until it reached a maximum at 50 mg SIF/kg feed (*P* < 0.05), and then it slowly decreased thereafter. The *CAT*, *GPx1a* and *GPx4b* mRNA levels reached a maximum at 50 mg SIF/kg feed and slowly decreased thereafter. The *GSTR*, *GSTP1* and *GSTP2* mRNA levels gradually increased until 50 mg SIF/kg feed and then decreased thereafter in grass carp. The *GSTO1*, *GSTO2* and *GR* mRNA levels increased until they reached a maximum at 50, 75 and 50 mg SIF/kg feed, respectively, and then they slowly declined thereafter. The *TOR* and *Nrf2* mRNA levels all peaked at the 50 mg SIF/kg feed. The *GPx1b*, *GPx4a* and S6K1 mRNA levels significantly increased until they reached a maximum level at 25 mg SIF/kg feed (*P* < 0.05), and then they slowly decreased thereafter. Conversely, the *Keap1a*, *Keap1b*, *4E-BP1* and *4E-BP2* mRNA levels reached a minimum value in the 50 mg/kg SIF group. The supplementation of SIF had no significant effect on the *CuZnSOD* mRNA level (*P* > 0.05).

**Fig 4:**
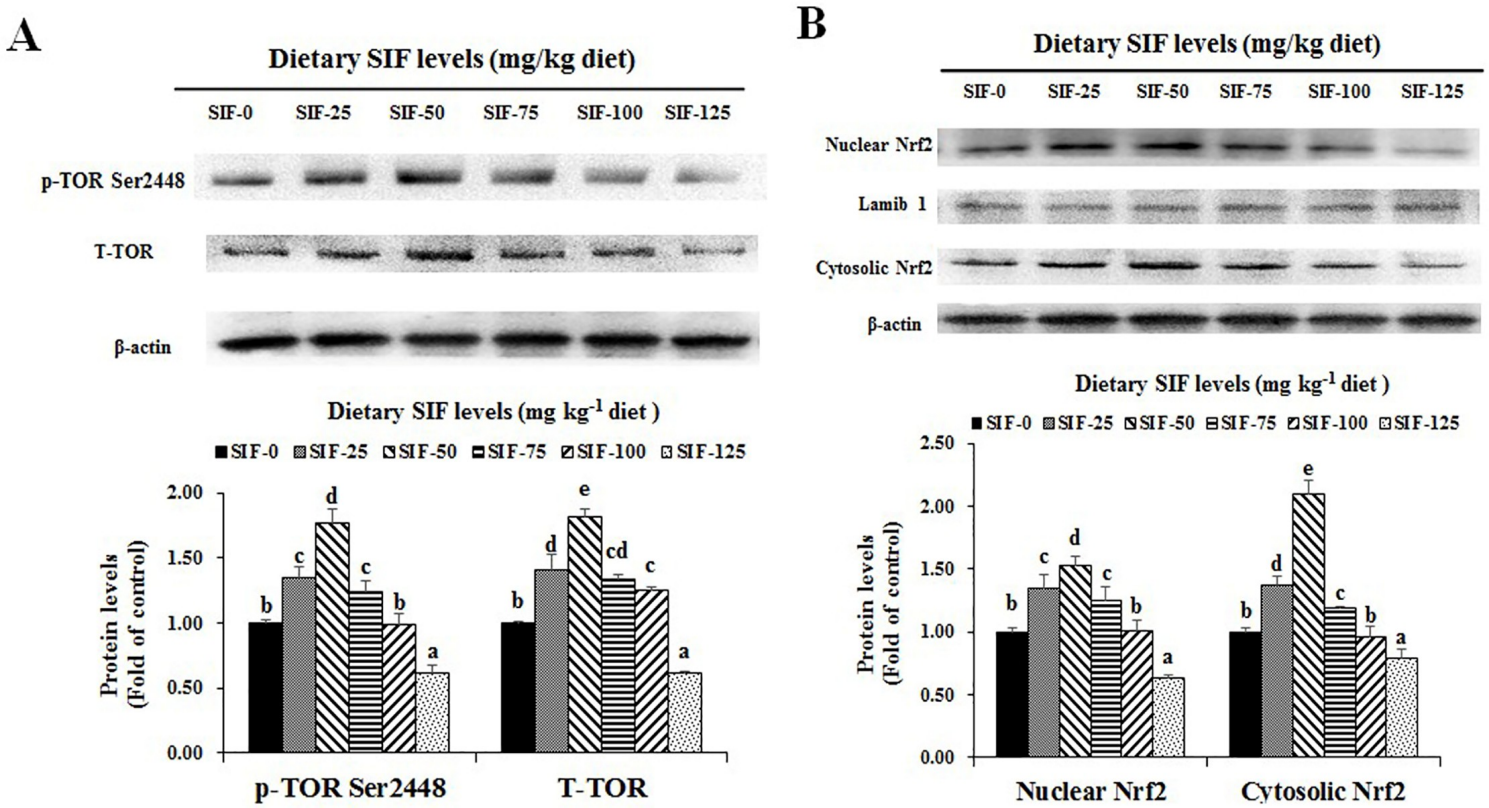
Effects of SIF on the protein levels of TOR and Nrf2 in the muscle of grass carp. **(A) p-TOR Ser2448 and T-TOR protein levels; (B) Nuclear Nrf2 and cytosolic Nrf2 protein levels.** Values are means (n = 6), error bars indicate SD, and different letters above a bar denote the significant difference between treatments (*P* < 0.05). p-TOR Ser2448, Phosphorylation on residue Ser2448 of TOR, T-TOR, total-TOR protein levels; Nuclear Nrf2, Nuclear Nrf2 protein levels; cytosolic Nrf2, cytosolic Nrf2 protein levels.

As presented in Fig 4A and 4B, SIF supplementation had a positive impact on TOR (target of rapamycin) and Nrf2 protein levels and on phosphorylation in fish muscle. Phosphorylation on residue Ser2448 of TOR (p-TOR Ser2448), the nuclear Nrf2 and cytosolic Nrf2 protein levels significantly differed among all treatments (*P* < 0.05) and were highest with the 50 mg/kg SIF treatment. The total TOR (T-TOR) protein level significantly increased until it reached a maximum at 50 mg SIF/kg feed (*P* < 0.05), and then it slowly declined thereafter.

## Discussion

In this study, we found that optimal dietary SIF supplementation (25 mg SIF/kg diet) improved the growth performance (PWG, SGR, FI and FE) of grass carp, which suggested that optimal dietary supplementation SIF could improve fish growth. Similar results had been observed in juvenile golden pompano (*Trachinotus ovatus*) [33]. It was reported that fish growth is tightly related to FI [41]. Our results demonstrated that the optimum dietary SIF increased FI, and correlation analysis showed the PWG of young grass carp was positively correlated with FI (R_PWG, FI_ = + 0.955, *P* < 0.01; R_SGR, FI_ = + 0.957, *P* < 0.01), indicating that SIF promoted feed intake thereby increasing fish growth. Fish muscle is the major edible part of fish, and the growth of fish primarily relies on muscle growth [42]. Thus, exploring the effect of SIF on meat quality is extremely important in fish. Fish meat quality can be evaluated by quality parameters, including nutritive quality, health benefits, flavour quality indicators and physical properties [14]. Hence, we surveyed the effects of SIF on these quality parameters and the potential mechanisms that influences these indices.

### Optimal dietary SIF supplementation improved the nutritive quality and health benefits of fish muscle

The protein and PUFA content can reflect the nutritive quality of fish meat [43]. In grass carp muscle, compared with the non-supplemented group, the protein content was significantly increased and obtained the maximum when fish fed diets with 50 mg/kg SIF, and PUFA level reached the maximum in the 50 mg/kg SIF group, and their content increased by 11.9% and 10.8%, respectively. These observation indicated that optimal dietary SIF supplementation improved the nutritive quality of fish meat. The increase in protein may be related to TOR signalling in fish muscle. Jiang et al. [40] showed that TOR signalling plays a key role in protein synthesis. As shown by our results, grass carp fed diets with 50 mg/kg SIF had significantly higher *TOR* mRNA level, total TOR and p-TOR Ser 2448 protein levels, indicating that hat increased protein by optimal dietary SIF supplementation might be partially related to the activation of TOR in fish muscle. However, the reasons for the increase in PUFA are unclear, which require further investigation.

The amount and type of FA can reflect the health benefits of fish [14]. Reducing the intake of SFA has been reported to decrease the risk of cardiovascular disease in humans [44]. Long chain n-3 PUFA, such as EPA and DHA, are thought to have health benefits for the human heart, brain and eyes [45]. Furthermore, it was reported that higher ratio of ∑n3/∑n6 reduced the risk of aggressive prostate cancer and cardiovascular disease in human [46]. Our results showed that, compared with the non-supplemented group, the total content of SFA was significantly decreased and reached the minimum and decreased by 12.1% in the 50 mg/kg SIF group; conversely, the EPA content significantly increased when fish fed with 25–75 mg/kg SIF, and reached the maximum and increased by 22.9% in 50 mg/kg SIF group. Meanwhile, we observed that, compared with the non-supplemented group, dietary SIF supplementation had no significant impact on DHA and ∑n3/∑n6 content, but the content obtained maximum and increased by 24.4% and 30% in 50 mg/kg group, respectively. These observation indicated that dietary SIF supplementation increased the health benefits of fish muscle. The increase in the EPA and DHA content might be partially associated with a-linolenic acid (ALA, 18:3n -3) and *Δ*6-D. Researchers have reported that ALA is a precursor to EPA and DHA [17]. *Δ6-D* was the rate-limiting step in the HUFA biosynthetic pathway, which includes EPA and EPA, and enzyme activity partly depends on *Δ6-D* gene expression in fish [17]. As shown by our results, optimal dietary SIF supplementation enhanced the ALA content and *Δ6-D* gene expression in fish muscle, supporting our hypothesis.

### Optimal dietary SIF supplementation improved the flavour quality indicators of fish muscle

FAA and 5'-IMP play important roles in the development of flavour and taste in animal meat [47,48]. The enhancement of the total FAA content could increase the taste and flavour intensity of beef [49]. We observed that, compared with the non-supplemented group, grass carp fed diets with 25–50 mg/kg SIF had significantly higher FAA content, and the total FAA level obtained the maximum and increased by 7% in the 50 mg/kg SIF group. Hence, our study implied that optimal dietary SIF supplementation could improve the taste and flavour of fish muscle. Moreover, umami taste is related to the induction and enhancement of the overall flavour perception of Yangtze *Coilia ectenes* meat [50]. Glu and 5'-IMP help create the palatable umami taste of meat, and the umami taste can be improved synergistically by the combination of umami amino acids and 5'-IMP in animal meat [51]. Our study demonstrated that, compared with the control group, Glu and 5'-IMP significantly increased when grass carp fed diets with 25–75 and 50 mg/kg SIF, respectively, and their content reached the maximum in the 50 mg/kg SIF group and increased by 29.0% and 13.1%, respectively. Additionally, an *in vitro* study showed that the interaction of several sweet amino acids (Ser, Gly and Ala) with 5'-IMP strongly enhanced the umami taste [52]. Our study demonstrated that, compared with non-supplemented group, Ser, Gly and Ala content significantly increased when fish fed with 50, 50 and 25–50 mg/kg SIF, respectively, and their content all reached the maximum in the 50 mg/kg SIF group and increased by 15.6%, 8.3% and 19.8% compared the control group, respectively. These observation implied that optimal dietary SIF supplementation could enhance the flavour quality indicators of fish meat. The SIF-increased total FAA content might be partially associated with insulin. Lee et al. [53] mentioned that increasing insulin might accelerate the uptake of blood FAA into muscle in Juvenile Sterlet Sturgeon (*Acipenser ruthenus*). An early study demonstrated that SIF could increase the serum insulin content of rats [25]. Nevertheless, the underlying mechanism responsible for these effects needs to be investigated further. Additionally, the SIF-increased 5'-IMP content might be partially related to Asp and Gly. Asp and Gly have been reported to be important substrates in the *de novo* synthesis pathway of purine nucleotides in Caco-2 cells [54]. Purine nucleotide supplementation increased the muscle 5'-IMP level in broiler [55]. Our study showed that the Asp and Gly content were the highest in the 50 mg/kg SIF group in grass carp, supporting our hypothesis.

### Optimal dietary SIF supplementation improved the physical properties of fish muscle

Physical properties, including WHC and tenderness, are important quality parameters of fish meat [14]. WHC can be evaluated by cooking loss, and a low cooking loss indicates high meat quality in fish [27]. Results showed that, optimal dietary SIF supplementation reduced the cooking loss, which was lower in the group fed the 50 mg/kg diet, indicating that SIF increased the fish muscle WHC. The SIF-elevated WHC might be partially ascribed to muscle collagen, cathepsin activity and apoptosis. An early study showed that increasing the collagen content and decreasing cathepsin activity elevated the WHC of fish muscle [14,56]. Generally, hydroxyproline quantification can be used to estimate the collagen content of Atlantic salmon (*Salmo salar*) [57]. Our results showed that, compared with non-supplemented group, dietary SIF supplementation (25–75 mg SIF/kg diet) increased the muscle hydroxyproline concentration and reduced the activities of cathepsin B and L, supporting our hypothesis. Additionally, research has demonstrated that inhibiting apoptosis can increase the WHC in ducks [29]. DNA fragmentation, a characteristic of apoptosis, involves caspases, including initiator caspases (*caspase-2*, *-8*, *-9*) and effector caspases (*caspase-3*, *-7*) in mice [58,59]. Our results showed that DNA fragmentation significantly decreased, and *caspase-2*, *-7 and caspase-*9 genes expression had a minimum in 25 mg SIF/kg group, and *caspase-3* as well as *caspase -8* obtained the minimum in 50 mg SIF/kg group. These observation showed that SIF-elevated the WHC might be associated with decreased apoptosis in fish muscle.

Tenderness can be evaluated by shear force, and a low shear force indicates high tenderness in Atlantic salmon [60]. We observed that optimal dietary SIF supplementation reduced the muscle shear force, which was lower in the group fed the 50 mg/kg diet, indicating that SIF supplementation increased the sensory tenderness of fish muscle. We speculated that the increased tenderness might be partially related to WHC and antioxidant capacity. An early study showed that a higher WHC often allows for lower shear force in fish muscle [60]. In this study, optimal dietary SIF supplementation enhanced WHC in fish muscle, supporting our hypothesis. In addition, research has reported that tenderness is partially reduced by lipid- and protein-oxidative damage in grass carp muscle [61]. GSH and antioxidant enzymes can reduce oxidative damage via scavenging ROS in grass carp [62]. In grass carp, antioxidant enzyme activities partially rely on their gene expression [14]. Our results showed that, dietary SIF supplementation (25–75 mg SIF/kg) reduced the MDA (lipid peroxidation product) and PC (protein oxidative product) content, and a higher level of SIF (50 mg SIF/kg) reduced the ROS content but enhanced the GST activity, meanwhile, a higher level of SIF supplementation (50–75 mg SIF/kg) increased MnSOD and GPx activities, and GR and CAT activities was increased when fish fed with 25–75 and 25–50 mg/kg SIF, respectively, and their genes expression were increased by an optimal SIF supplementation, respectively. These observation indicated that optimal dietary SIF-improved tenderness might be ascribed to less oxidative damage and an increase in the GSH content and antioxidant enzyme activity regulated by SIF-increased antioxidant enzyme genes (except *CuZnSOD*). Antioxidant enzyme mRNA levels can be partially modulated by the Nrf2 signalling pathway [62]. Nrf2 nuclear translocation plays a vital role in activating the Nrf2 signalling pathway [40], which can be evaluated by the nuclear Nrf2 protein level in mouse livers [63]. In this study, optimal dietary SIF supplementation (50 mg SIF/kg) elevated the mRNA, nuclear protein and cytosolic protein levels of *Nrf2*, indicating that optimal dietary SIF-enhanced antioxidant enzyme gene expression (except *CuZnSOD*) can be regulated by an increase in *Nrf2* nuclear translocation in fish muscle. Furthermore, decreasing *Keap1*gene expression, an Nrf2-binding protein, can facilitate *Nrf2* nuclear translocation in grass carp [40]. In our research, optimal dietary SIF supplementation depressed the *Keap1a* and *1b* mRNA levels, indicating that optimal dietary SIF increased *Nrf2* nuclear translocation and increased antioxidant enzyme gene expression via decreasing *Keap1* gene expression in fish muscle.

Optimal dietary SIF supplementation increased the physical properties of fish muscle. The dietary SIF-increased WHC was mainly ascribed to the attenuation of cathepsin activity and apoptosis and to the elevation of the collagen content in fish muscle. The dietary SIF-increased tenderness was mainly ascribed to an increased WHC and antioxidant capacity regulated by *Nrf2* signalling in fish muscle.

### Excessive SIF decreased flesh quality in fish

From the data, we found that compared with optimal SIF supplementation (the 25 or 50 mg SIF/kg diet), excessive SIF (100 or 125 mg/kg diet) caused decreases in protein, lipid, EPA, PUFA, total amino acid, hydroxyproline content; and increases in cooking loss, cathepsin B and L activities in the fish muscle; these findings indicated that excessive SIF depressed the flesh quality of fish. As mentioned above, protein synthesis was tightly related to TOR signaling. We further observed that, compared with optimal SIF supplementation, high levels of dietary SIF depressed the TOR mRNA level, total TOR and p-TOR Ser 2448 protein level, which indicated that excessive SIF could decreased the protein which was partly associated with inhibiting TOR signalling by SIF excess. Meanwhile, compared with optimal SIF supplementation, excess SIF caused increases in lipid peroxidation and protein oxidation; but decreased GSH content, antioxidant enzymes activity and their genes expression (except CuZnSOD), which indicted that excessive SIF could induce oxidative damage which was partly associated with decreasing GSH content and enzymes activity (except CuZnSOD). At the same time, excessive dietary SIF supplementation (100–125 mg SIF/kg diet) up-regulated the mRNA levels of Keap1 and down-regulated the cytosolic and nuclear Nrf2 protein levels, which suggests that SIF excess could partly inhibit Nrf2 signalling. The mechanisms by which excessive SIF has these impacts are still indistinct, but may be partly associated with the pro-oxidant effects of excess SIF. In pig, excessive SIF had pro-oxidant function, which could enhance ROS level in pig [64]. Previous study reported that a high level of ROS could increase the shear force [65], decrease the collagen, protein and lipid synthesis resulting in the decline of meat quality in animal [66]. Hence, we speculated that excessive SIF decrease the flesh quality of fish might be partly related to SIF-increased ROS content, which need further investigation.

## Conclusions

**Fig 5:**
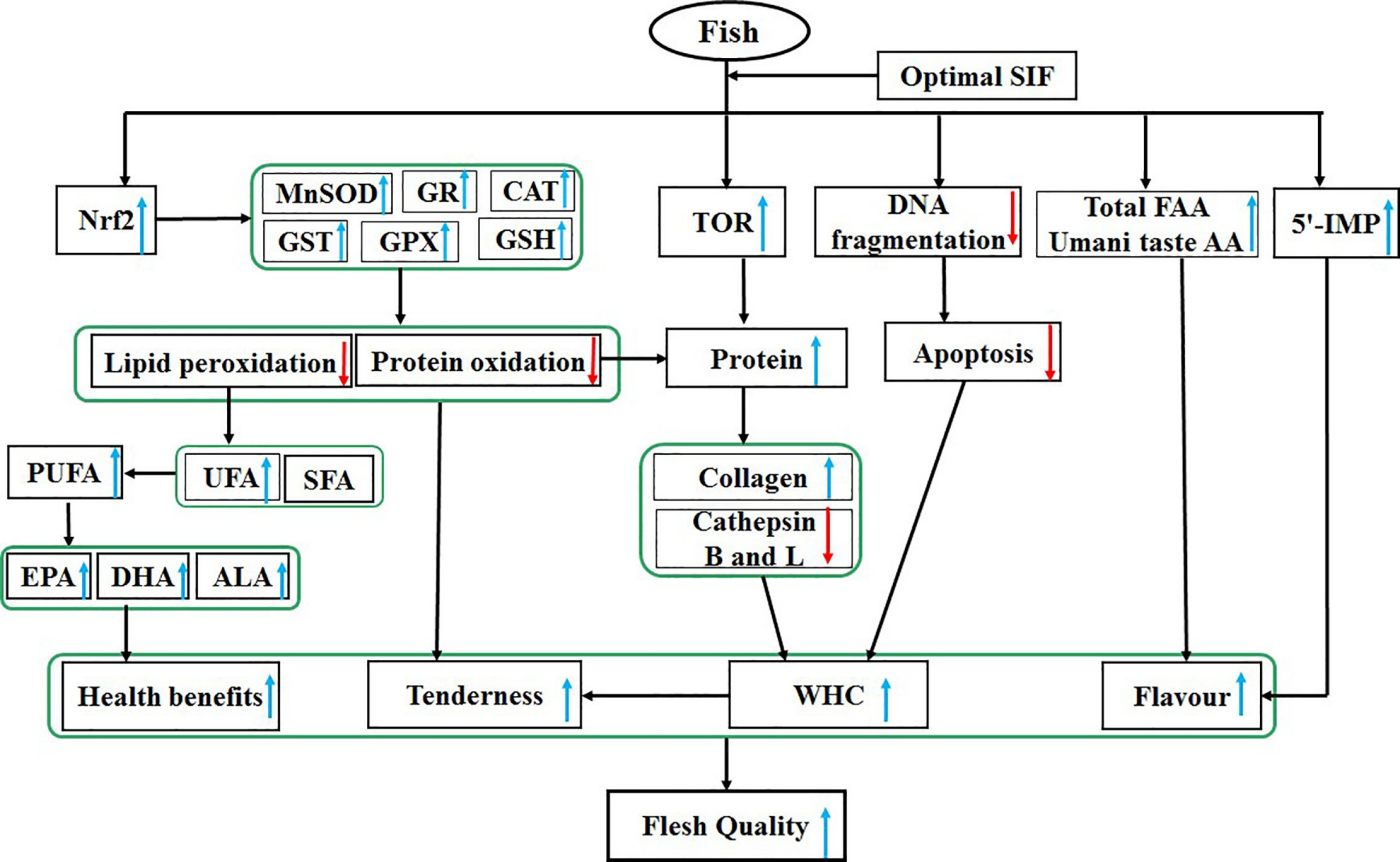
General summary for the effects of dietary SIF on meat quality and its potential signaling pathways in the muscle of fish. WHC, water-holding capacity; Nrf2, NF-E2-related factor 2; 5'-IMP, 5'-inosine monophosphate; FAA, free amino acids; TOR, target of rapamycin.

As shown in Fig 5, the research showed that optimal dietary SIF supplementation had a potential to improve the meat quality of grass carp. (1) Optimal dietary SIF supplementation (the 25 or 50 mg SIF/kg diet) increased muscle protein total PUFA, healthcare fatty acid (ALA, EPA and DHA), total FAA, Glu, Asp, 5'-IMP content, WHC and tenderness, promoting the nutritive value, health benefits, flavour quality indicators in the muscle of fish; (2) Optimal dietary SIF supplementation (the 25 or 50 mg SIF/kg diet) increased WHC and tenderness, enhancing the meat quality. Further exploration showed that the SIF-elevated WHC might be associated with a high collagen concentration and low cathepsin B and L activities as well as reduced apoptosis in fish muscle, whereas SIF-increased tenderness may be associated with an elevated WHC and an enhanced antioxidant capacity in fish muscle. On the other hand, SIF-increased antioxidant capacity was ascribed to an increase in the GSH content and antioxidant enzyme (except CuZnSOD) activities regulated by their gene expression and Nrf2 signalling in fish muscle. (3) Excessive SIF (100 or 125 mg/kg) could inhibit some of these positive effects, when compared with the optimal level of SIF.
